# Multiplex LNA probe-based RAP assay for rapid and highly sensitive detection of rifampicin-resistant *Mycobacterium tuberculosis*

**DOI:** 10.3389/fmicb.2023.1141424

**Published:** 2023-04-27

**Authors:** Ruiqing Zhang, Xichao Ou, Xiuli Sun, Guohao Fan, Bing Zhao, Fengyu Tian, Fengyu Li, Xinxin Shen, Yanlin Zhao, Xuejun Ma

**Affiliations:** ^1^National Institute for Viral Disease Control and Prevention, Chinese Center for Disease Control and Prevention, Beijing, China; ^2^National Center for Tuberculosis Control and Prevention, Chinese Center for Disease Control and Prevention, Beijing, China; ^3^Clinical Laboratory, North China University of Science and Technology, Tangshan, China; ^4^Hebei Key Laboratory of Molecular Medicine, Hebei Medical University, Shijiazhuang, China; ^5^Center for Biosafety Mega-Science, Chinese Academy of Sciences, Wuhan, China

**Keywords:** multiplex, RAP, rifampicin-resistant tuberculosis (RR-TB), rapid, highly sensitive

## Abstract

**Objectives:**

The World Health Organization (WHO) Global tuberculosis Report 2021 stated that rifampicin-resistant tuberculosis (RR-TB) remains a major public health threat. However, the in-practice diagnostic techniques for RR-TB have a variety of limitations including longer time, lack of sensitivity, and undetectable low proportion of heterogeneous drug resistance.

**Methods:**

Here we developed a multiplex LNA probe-based RAP method (MLP-RAP) for more sensitive detection of multiple point mutations of the RR-TB and its heteroresistance. A total of 126 clinical isolates and 78 sputum samples collected from the National Tuberculosis Reference Laboratory, China CDC, were tested by MLP-RAP assay. In parallel, qPCR and Sanger sequencing of nested PCR product assay were also performed for comparison.

**Results:**

The sensitivity of the MLP-RAP assay could reach 5 copies/μl using recombinant plasmids, which is 20 times more sensitive than qPCR (100 copies/μl). In addition, the detection ability of rifampicin heteroresistance was 5%. The MLP-RAP assay had low requirements (boiling method) for nucleic acid extraction and the reaction could be completed within 1 h when placed in a fluorescent qPCR instrument. The result of the clinical evaluation showed that the MLP-RAP method could cover codons 516, 526, 531, and 533 with good specificity. 41 out of 78 boiled sputum samples were detected positive by MLP-RAP assay, which was further confirmed by Sanger sequencing of nested PCR product assay, on the contrary, qPCR was able to detect 32 samples only. Compared with Sanger sequencing of nested PCR product assay, both the specificity and sensitivity of the MLP-RAP assay were 100%.

**Conclusion:**

MLP-RAP assay can detect RR-TB infection with high sensitivity and specificity, indicating that this assay has the prospect of being applied for rapid and sensitive RR-TB detection in general laboratories where fluorescent qPCR instrument is available.

## Introduction

The World Health Organization (WHO) Global tuberculosis Report 2021 stated that rifampicin-resistant tuberculosis (RR-TB) remains a major public health threat (World Health Organization [WHO], 2021). RR-TB is mainly linked to the mutations of the 81-bp rifampin resistance-determining region (RRDR) of the rpoB gene, corresponding to 27 amino acids or codons. Moreover, the mutations of codons 516, 526, 531, and 533 in RRDR are the most common, accounting for 75–90% of RIF resistance (Huang et al., 2002; Liu et al., 2022). The early, rapid, and sensitive diagnosis of RR-TB can certainly help doctors choose effective antituberculosis drugs for patients and prevent the further spread of TB.

However, conventional culture-based drug susceptibility test requires several weeks to identify slow-growing drug-resistant *Mycobacterium tuberculosis* (MTB) (Walzl et al., 2018). Recently, nucleic acid amplification techniques (NAATs) have been progressively used for the diagnosis of RR-TB (Machado et al., 2019; Acharya et al., 2020). Among them, the locked nucleic acid (LNA) probe real-time PCR assay is one of the most reliable and specific approaches. The LNA probe can easily discriminate one-base mutation through the amplification curves, without complex determinations or additional Tm analyses (You et al., 2006; Zhao et al., 2015; Peng et al., 2016). Nonetheless, the LNA probe-based PCR assays are time-consuming and need to be improved in sensitivity.

Previously, our laboratory reported a novel Recombinase Aided PCR (RAP) method for rapid and highly sensitive detection of respiratory viruses (Fan et al., 2021). The principle of the RAP method involves enriching tiny amounts of target DNA fragments using recombinase-aided amplification (RAA) (Ma, 2022) for 10 min followed by amplifying the enriched templates using qPCR with fewer thermal cycles. In this study, we developed a multiplex LNA probe-based RAP method (MLP-RAP) for the first time to detect multiple point mutations of the RR-TB. Furthermore, the docosane-based physical isolation strategy allows both RAA and qPCR reactions carried out in a single tube within 1 h. Therefore, MLP-RAP enabled rapid and highly sensitive detection of rifampicin-resistant *M. tuberculosis*.

## Materials and methods

### Strains and specimens

The standard *Mycobacterium tuberculosis* strain (H37Rv) and 20 strains of MTB resistant or sensitive to rifampicin (RIF) were provided by the National Tuberculosis Reference Laboratory, Chinese Center for disease control and prevention (CDC). The MTB H37Rv standard strain and 10 RIF-susceptible strains showed no mutations in the RRDR region of the rpoB gene, and 10 RIF-resistant strains contained 10 types (516-GTC, 516-TAC, 526-GAC, 526-TAC, 526-CGC, 526-CTC, 526-AAC, 531-TTG, 531-TGG, and 533-CCG) of rpoB mutations, covering the most frequent mutations at codons 516, 526, 531, and 533.

The specimens used for clinical evaluation included 126 clinical isolates and 78 sputum samples, all of which were obtained from the National Tuberculosis Reference Laboratory, China CDC. In addition, 126 clinical isolates of *M. tuberculosis* with well-defined information on Phenotypic drug susceptibility test (DST), GeneXpert MTB/RIF (Cepheid, USA) result, and Whole Genome Sequencing (WGS) were collected for the present study. The details of the three methods (Phenotypic DST and WGS) are described in Liu et al. (2022).

This study was performed with the approval of the Center for Disease Control and Prevention of China. This study obtained written informed consent from patients.

### DNA extraction

DNAs of MTB H37Rv standard strain and 20 strains of MTB resistant or sensitive to RIF were extracted by the boiling method: 100 μl of bacterial suspension was boiled at 100°C for 15 min. After cooling, the suspension was centrifuged at 12,000 × *g* for 5 min, and 50 μl of the supernatant containing DNA was transferred into a new microcentrifuge tube for specificity evaluation of the MLP-RAP assay.

Both 126 clinical isolates and 78 sputum samples were extracted by the boiling method: 500 μl of the sample was heated at 100°C for 15 min, ultrasonicated for 15 min, centrifuged at 13,000 × *g* for 5 min, and the supernatant was used as a DNA template for clinical performance of the MLP-RAP assay.

### Recombinant plasmids preparation

The recombinant plasmid pUC57 harboring a 960 bp of the rpoB gene including 81 bp of RRDR, from 760647 to 761606 nt of the MTB H37Rv reference strain (AL123456.3) was synthesized by Tsingke Biotechnology Co., Ltd., Beijing, China. Five types of recombinant plasmids were prepared: one wild type (WT), four mutant types covering codon 516 (D516G), codon 526 (H526Y and H526D), and codon 531 (S531L). Then, each of these recombinant plasmids was diluted ten-fold from 10^8^ to 10^0^ copies/μl and used as standards for the evaluation of the analytical performance of the MLP-RAP assay.

### The principle of MLP-RAP assay

Multiplex LNA probe-based RAP method combined the advantages of both RAP and LNA Probe-Based PCR was performed in a conventional qPCR device. The principle of RAP involves a first-round amplification step using one pair of RAA primers (outer primers) followed by a second round of amplification using one pair of qPCR primers (inner primers) and qPCR probes (Fan et al., 2021). The qPCR probes were modified with LNA, ensuring the distinguished base-mutation recognition ability (Owczarzy et al., 2011).

**FIGURE 1:**
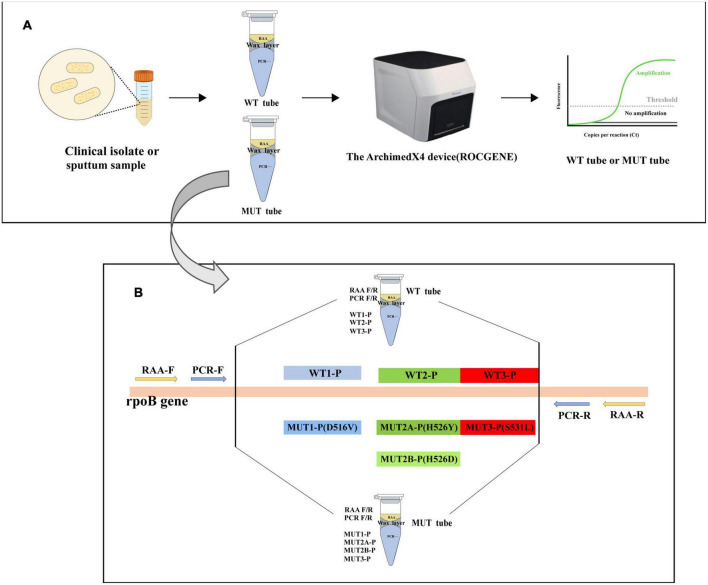
**(A)** Flow chart of the MLP-RAP method. **(B)** The schematic diagram of the MLP-RAP method for detection of RR-TB. RAA primers, qPCR primers, and qPCR LNA probes in WT and MUT tubes.

The schematic diagram of the MLP-RAP assay for detecting rifampicin-resistant *Mycobacterium tuberculosis* was shown in Figure 1. The primer and probe set for MLP-RAP assay of RR-TB included RAA-F, RAA-R, PCR-F, PCR-R, WT1-P, WT2-P, WT3-P, MUT1-P, MUT2A-P, MUT2B-P, and MUT3-P. We designed two tubes, referred to as wild type (WT) tube and mutant type (MUT) tube, for simultaneous detection of rifampicin-susceptible and rifampicin-resistant TB in each sample. The RAA primers (RAA-F and RAA-R) and PCR primers (PCR-F and PCR-R) were the same for each tube, but the LNA probes were base-specific (different) in WT and MUT tubes. The WT tube had three LNA probes labeled with different fluorescence, namely WT1-P with HEX label covering codon 516, WT2-P with Cy5 label covering codon 526, and WT3-P with FAM label covering codons 531 and 533. In the MUT tube, there were four differently labeled fluorescent LNA probes (MUT1-P with HEX label, MUT2A-P with Cy5 label, MUT2B-P with ROX label, and MUT3-P with FAM label). The four LNA probes were exactly matching four mutant rpoB gene sequences (D516V, H526Y, H526D, and S531L), respectively, which could accurately identify the presence of mutation types and cover the base mutation positions in mutant rpoB gene sequences.

Multiplex LNA probe-based RAP method assay was conducted in two tubes (WT and MUT). In each tube, the RAA reaction was performed at 39°C in the first stage, docosane formed a wax layer to perfectly separate the RAA (upper phase) from the qPCR systems (lower phase). When the RAP turned into the qPCR stage at 95°C, the wax layer with a melting point of 43–46°C melted and moved to the top layer of the tube. Meanwhile, the RAA components were inactivated at 95°C and mixed with the PCR system. The RAA-amplified DNA fragment was therefore used as a template for qPCR.

### MLP-RAP assay protocol

Our previous study demonstrated that RAP achieved the best amplification efficiency in the ratio of 1:4 (10 μl RAA mix versus 40 μl qPCR mix). In the MLP-RAP assay, the RAA reactions were the same for each WT and MUT tube, only the LNA probes of qPCRs were different in WT and MUT tubes. A total of 30 μl wax layer of docosane was placed between the RAA and PCR systems. In WT tubes, the RAA reaction (total 10 μl) comprised 14 mM of magnesium ion (Mg^2+^), 0.5 μM of each RAA primer, 1 μl of plasmid template with various concentrations or 2 μl extracted DNA, and RAA reaction buffer (Jiangsu, Qitian, Bio-tech Co. Ltd.). The qPCR (total 40 μl) contained 1 μM of each PCR primer, 0.3 μM of WT1-P, 0.2 μM of WT2-P, 0.4 μM of WT3-P, 0.12 mM of dNTP, and qPCR buffer (Entrans qPCR Probe Set V2, ABclonal, Wuhan, China). In MUT tubes, the qPCR contained 0.3 μM of MUT1-P, 0.6 μM of MUT2A-P, 0.2 μM of MUT2B-P, and 0.6 μM of MUT3-P. The WT and MUT tubes were then transferred to the ArchimedX4 device (ROCGENE). The MLP-RAP procedure was as follows: 39°C for 10 min, 95°C for 5 min, 24 cycles at 95°C for 15 s, 60°C for 30 s, and 72°C for 30 s, and the results could be observed in real-time. Each run contained a negative control (water).

**FIGURE 2:**
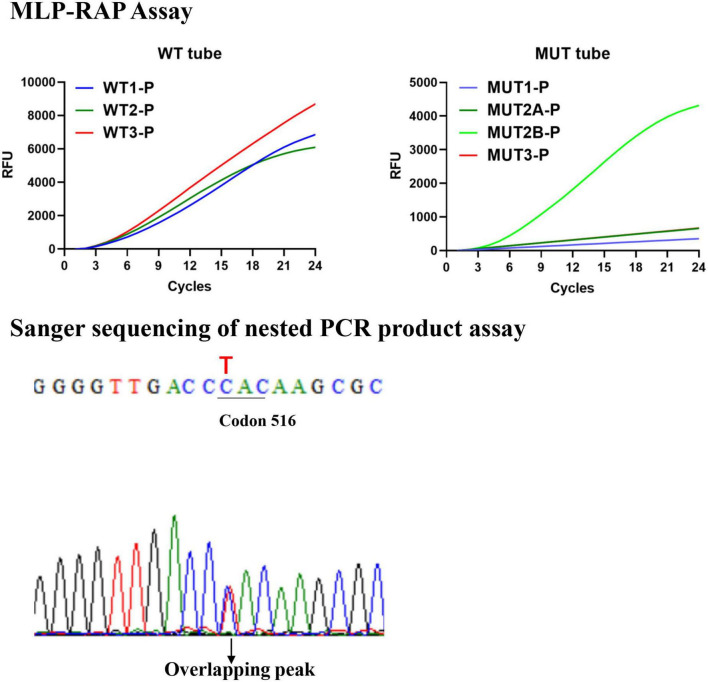
The results of 526-TAC (rpoB_H445Y) heteroresistance clinical isolate by the MLP-RAP and Sanger sequencing methods.

Multiplex LNA probe-based RAP method result interpretation: The outcome needs to be combined with the results of the WT and MUT tubes. There are three possible results: (1) Negative: No curve for all channels of the WT and MUT tubes. (2) No indication of resistance (sensitive): all three channels of the WT tube have positive curves and no curve for the four channels of the MUT tube. (3) Resistant: the specific types of resistance at codons 516, 526, 531, or 533 (see Table 1), or other resistant cases. Take Figure 2 for example, a clinical isolate with 526-TAC heteroresistance, according to Table 1, the conclusion is that this isolate is codon 516 sensitive, codon 526 sensitive, 526-TAC heteroresistance, and codon 531 or 533 sensitive.

**TABLE 1:** Interpretation of MLP-RAP results.

Codon 516		WT tube-WT1 channel	
		**+**	**–**
MUT tube-MUT1 channel	**+**	516-GTC heteroresistance	516-GTC resistance
	**–**	Codon 516 sensitive	Codon 516 resistance
**Codon 526**		**WT tube-WT2 channel**	
		**+**	**–**
MUT tube-MUT2A channel	**+**	526-GAC heteroresistance	526-GAC resistance
	**–**	Codon 526 sensitive	Codon 526 resistance
MUT tube-MUT2B channel	**+**	526-TAC heteroresistance	526-TAC resistance
	**–**	Codon 526 sensitive	Codon 526 resistance
**Codon 531 or 533**		**WT tube-WT3 channel**	
		**+**	**–**
MUT tube-MUT3 channel	**+**	531-TTG heteroresistance	531-TTG resistance
	**–**	Codon 531 or 533 sensitive	Codon 531 or 533 resistance

### Analytical sensitivity and specificity of the MLP- RAP assay

We evaluated the specificity of the MLP-RAP assay using MTB H37Rv standard strain and 20 strains of MTB resistant or sensitive to RIF per the MLP-RAP assay protocol.

The sensitivity of the MLP-RAP assay for detecting RR-TB was determined using WT plasmid and four MUT plasmids (D516V, H526D, H526Y, and S531L). A panel of diluted five recombinant plasmids (10^6^, 10^5^, 10^4^, 10^3^, 10^2^, 10, and 5 copies/μl) was tested to ascertain the endpoint dilution and eight replicates were performed.

### Sensitivity of the MLP- RAP assay for rifampicin heteroresistance

To investigate the ability of the MLP-RAP assay to detect less-abundant mutants in mixed templates, we prepared four series of mixtures containing 0, 5, 10, 15, 25, 50, 75, and 100% mutant DNA (corresponding to D516V, H526D, H526Y, and S531L individually) at the concentration of 10^4^ *M. tuberculosis* copies/μl. Further, we attempted to analyze the MLP-RAP detectability limit for rifampicin heteroresistance. Then, using S531L as an example, we prepared templates with a series of concentrations containing 0, 5, 10, 15, 25, 50, 75, and 100% of the mutation percentage at two different DNA concentration (5 × 10^3^ and 5 × 10^2^ copies/μl). The mixed templates were then tested for eight replicates by MLP-RAP detection.

### The qPCR assay and sanger sequencing of nested PCR products

The qPCR assay also had WT and MUT tubes like the MLP-RAP assay. In the WT tube, the qPCR (total 50 μl) contained 1 μM of each PCR primer, 0.3 μM of WT1-P, 0.2 μM of WT2-P, 0.4 μM of WT3-P, 0.12 mM of dNTP, 3 mM of MgCl_2_, and qPCR buffer (Entrans qPCR Probe Set V2, ABclonal, Wuhan, China). In the MUT tube, the qPCR contained 0.3 μM of MUT1-P, 0.6 μM of MUT2A-P, 0.2 μM of MUT2B-P, and 0.6 μM of MUT3-P. The WT and MUT tubes of qPCR assay were then transferred to the ArchimedX4 device (ROCGENE). The qPCR procedure was as follows: 95°C for 5 min, 45 cycles at 95°C for 15 s, 60°C for 30 s, and 72°C for 30 s, and the results could be observed in real-time. Each run contained a negative control (water).

The primers for the first round of nested PCR were TB-F (CTTGCACGAGGGTCAGACCA) and TB-R (ATCTCGTCGCTAACCACGCC) from a published article (Xue-jun, 2013), and the amplification length of the first round PCR was 543 bp. In addition, the primers for the second round of nested PCR were RAA-F (AGGACGTGGAGGCGATCACACCGCAGACGTT) and RAA-R (CAGGGGTTTCTATCGGGCACATCCGGCCGTA) from MLP-RAP, and the amplification length of the second round PCR was 249 bp. Nested PCR reactions were amplified using the Entrans qPCR Probe Set V2 kit (ABclonal). The nested PCR assay was then transferred to the ArtGene (TM) A300 device (LongGene). The working condition of two rounds of the nested PCR assay was the same, as follows: 95°C for 10 min, 45 cycles at 95°C for 15 s, 60°C for 34 s, and 72°C for 34 s, and each run contained a negative control (water). Nested PCR products were sent to Sangon Biotech for Sanger sequencing.

### Clinical performance of the MLP-RAP assay

Both 126 clinical isolates and 78 sputum samples were used for the clinical performance of the MLP-RAP assay. For comparison, qPCR and Sanger sequencing of nested PCR products assays were carried out as references in parallel as described previously. Statistical analysis was performed with IBM SPSS Statistics, version 21 (IBM Corporation, NY, USA). Kappa and McNemar’s tests were used to analyze the results of the clinical performance of MLP-RAP, qPCR, and Sanger sequencing of nested PCR products assay. When the *P*-value was less than 0.05, the results were considered statistically significant. In addition, a total of 78 sputum specimens were tested in parallel using GeneXpert MTB/RIF (Cepheid, USA) according to the operation manual.

## Results

### Analytical performance of MLP-RAP assay

The specificity results demonstrated that the MLP-RAP method possessed good specificity for rifampicin-resistant or sensitive strains of *M. tuberculosis*.

**FIGURE 3:**
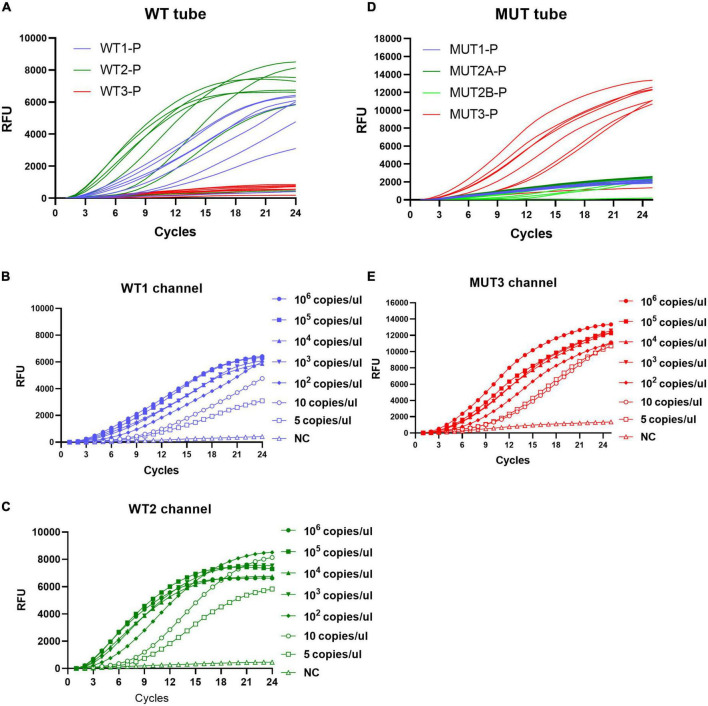
Sensitivity of the MLP-RAP assay using diluted S531L plasmid for eight replicates (10^6^, 10^5^, 10^4^, 10^3^, 10^2^, 10, and 5 copies/μl). In WT tube **(A)**, WT1 channel **(B)** and WT2 channel **(C)** have signals. In MUT tube **(D)**, MUT3 channel **(E)** has a signal. The sensitivity of the WT1 channel, WT2 channel, and MUT3 channel could reach 5 copies/μL.

As shown in Figure 3, taking S531L as an example, mutations existed at codon 531 of RRDR, which inhibit hybridization with WT3-P of the WT tube and lighten the signal of the MUT3-P of the MUT tube, and the sensitivity of MLP-RAP assay was 5 copies/μl. Similarly, the sensitivity of the other four plasmids could reach 5 copies/μl, respectively (Supplementary Figures 1–4). MLP-RAP assay demonstrated the detection limit of 5 copies/μl for rifampicin resistance, which is lower than those of qPCR (100 copies/μl). The qPCR results were not shown.

### Sensitivity of the MLP- RAP assay for rifampicin heteroresistance

**FIGURE 4:**
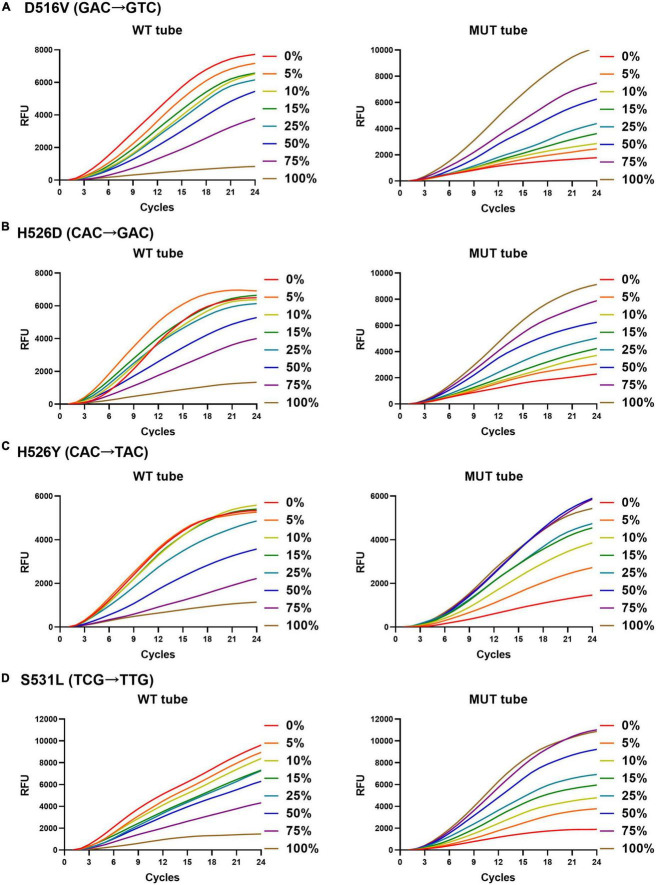
MLP-RAP assay for detecting four mutation types at a total concentration of 1 × 10^4^ copies/μl for eight replicates, D516V **(A)**, H526D **(B)**, H526Y **(C)**, and S531L **(D),** respectively. In panel **(A)**, the WT tube only shows the result of the WT1 channel, and the MUT tube only shows the result of the MUT1 channel. In panel **(B)**, the WT tube only shows the result of the WT2 channel, and the MUT tube only shows the result of the MUT2A channel. In panel **(C)**, the WT tube only shows the result of the WT2 channel, and the MUT tube only shows the result of the MUT2B channel. In panel **(D)**, the WT tube only shows the result of the WT3 channel, and the MUT tube only shows the result of the MUT3 channel.

**FIGURE 5:**
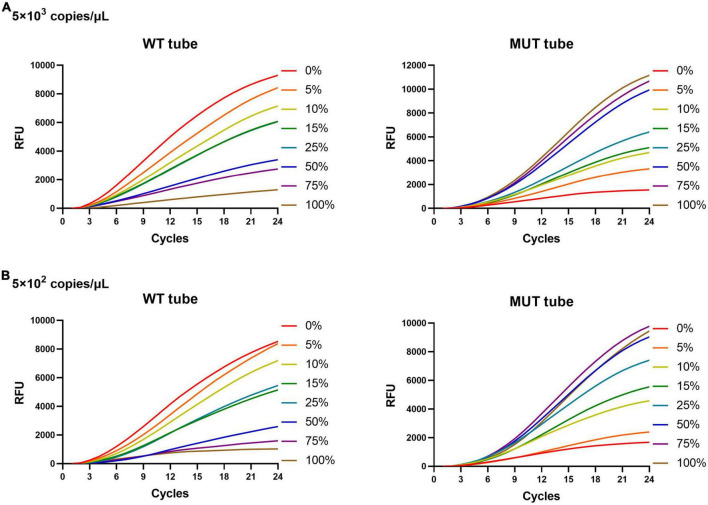
MLP-RAP assay for detecting S531L (TCG→TTG) type plasmid of two different total DNA concentrations for eight replicates, 5 × 10^3^ copies/μl **(A)** and 5 × 10^2^ copies/μL **(B),** respectively. In panels **(A,B)**, the WT tube only shows the result of the WT3 channel, and the MUT tube only shows the result of the MUT3 channel.

The rifampicin heteroresistance detection ability of the MLP-RAP assay was evaluated using mixed plasmids in different proportions. The results showed that the MLP-RAP assay had a mutation detection capacity of 5% for a mixed template of four mutation types at a total concentration of 10^4^ copies/μl, as shown in Figure 4. Furthermore, take S531L for example, the results showed that the MLP-RAP assay still had a mutation detection capacity of 5% at the total concentration of 5 × 10^3^ and 5 × 10^2^ copies/μl, as shown in Figure 5.

### Clinical performance of MLP-RAP assay

A total of 126 clinical isolates were detected, and the MLP-RAP results showed that 39 samples were no indication of resistance, 81 samples were resistant and 6 samples were negative, of which 120 were detected by qPCR with the Ct range from 16 to 30. Among the 81 resistant samples, 6 were 526-TAC (rpoB_H445Y) heteroresistance and 1 was 531-TTG (rpoB-S450L) heteroresistance detected by MLP-RAP, which were not detected by GeneXpert as shown in Table 2. Six negative samples by the MLP-RAP assay were four 511-CCG (rpoB-L430P) and two 513-AAA (rpoB-Q432L) resistant samples by WGS. Therefore, the mutation types of codon 511 and codon 513 cannot be covered by MLP-RAP assay. One sample with the result of 516-GTC resistance by the MLP-RAP assay did not match the result (sensitive) of phenotypic DST. It should be noted that the resistance results determined by drug resistance genes and the phenotypic drug sensitivity methods sometimes did not agree exactly, but the MLP-RAP method was accurate at the gene detection level of drug resistance. The remaining 119 samples showed consistent results among the MLP-RAP, Phenotypic DST, and WGS.

**TABLE 2:** Phenotypic DST, WGS, and MLP-RAP results of 126 clinical isolates.

Sort	Phenotypic DST	WGS	GeneXpert result	MLP-RAP	Sample size
1	S	S	S	No indication of resistance	39
2	S	516-GTC (rpoB-D435V)	R (probe B)	516-GTC resistance	1
3	R	516-TAC (rpoB-D435Y)	R (probe B)	Codon 516 resistance	3
4	R	526-GAC (rpoB-H445D)	R (probe D)	526-GAC resistance	7
5	R	526-TAC (rpoB-H445Y)	R (probe D)	526-TAC resistance	6
6	R	526-CGC (rpoB-H445R)	R (probe D)	Codon 526 resistance	2
7	R	526-CTC (rpoB-H445L)	R (probe D)	Codon 526 resistance	5
8	R	526-AAC (rpoB-H445N)	R (probe D)	Codon 526 resistance	2
9	R	531-TTG (rpoB-S450L)	R (probe E)	531-TTG resistance	39
10	R	531-TGG (rpoB-S450W)	R (probe E)	Codon 531 or 533 resistance	1
11	R	533-CCG (rpoB-L452P)	R (probe E)	Codon 531 or 533 resistance	8
12	R	526-TAC (rpoB-H445D)	S	526-TAC heteroresistance	6
13	R	531-TTG (rpoB-S450L)	S	531-TTG heteroresistance	1
14	R	511-CCG (rpoB-L430P) or 513-AAA (rpoB-Q432L)	R (probe A or probe B)	N	6

DST, drug susceptibility test; R, resistant; S, sensitive; N, negative.

The MLP-RAP results of all clinical isolates were consistent with Sanger sequencing of nested PCR products assay with 100% concordance. Sanger sequencing of nested PCR products assay resulted in an overlapping peak at the first base (T/C) of Codon 526, which verified six 526-TAC (rpoB_H445Y) heteroresistance clinical isolates detected by MLP-RAP. Figure 2 showed one of the six isolates using these two methods. Similarly, Sanger sequencing verified one 531-TTG heteroresistance clinical isolate detected by MLP-RAP.

A total of 78 boiled sputum samples were tested by the MLP-RAP assay, of which 41 showed no indication of resistance (sensitive) and 37 were negative. MLP-RAP detected 41 positive samples (sensitive), of which 32 were detected by qPCR with the Ct range from 27 to 35 as shown in Table 3. Additionally, the results of the MLP-RAP and the qPCR assays were analyzed statistically by the Kappa test. The Kappa value was 0.771 (*P* < 0.001) with significant differences among them. Compared with Sanger sequencing of nested PCR product assay, both the specificity and sensitivity of the MLP-RAP assay using 78 sputum samples were 100%. In addition, the MLP-RAP results were consistent with the GeneXpert results by 100% concordance.

**TABLE 3:** The clinical performance of the MLP-RAP with 78 sputum samples versus results of qPCR and sanger sequencing of nested PCR product assays.

		MLP-RAP		Performance characteristics		
		Positive	Negative	Sensitivity (%)	Specificity (%)	Kappa
qPCR	Positive	32	0	78.05	100	0.771
	Negative	9	37			
	Total (*n* = 78)	41	37			
Sanger sequencing	Positive	41	0	100	100	1.000
	Negative	0	37			
	Total (*n* = 78)	41	37			

## Discussion

The detection capability of current molecular assays is not sufficient in detecting RR-TB samples with lower concentration, therefore we need an ultra-sensitive method. In addition, the problem of heterogeneous resistance to RR-TB in the clinical setting has become increasingly prominent (Hofmann-Thiel et al., 2009; Operario et al., 2017). However, the detection of heteroresistance is challenging using current molecular assays. Here we developed the MLP-RAP method with WT and MUT tubes for more sensitive detection of RR-TB and its heteroresistance. The result of the clinical evaluation showed that the MLP-RAP method could cover the rifampicin-resistant mutations at codons 516, 526, 531, and 533 with good specificity, and the 526-TAC (rpoB_H445Y) and 531-TTG (rpoB-S450L) heteroresistance could be detected. Theoretically, all four heteroresistance (516-GTC, 526-GAC, 526-TAC, and 531-TTG) can be detected. The reaction time of the MLP-RAP method was greatly reduced, which could be completed within 1 h in a conventional fluorescent qPCR instrument. During the COVID-19 pandemic, provinces, and cities centers for disease control and prevention (CDC) in China have been well-equipped with qPCR instruments and skillful staff. Additionally, the MLP-RAP assay for sputum samples has low requirements (boiling method) for nucleic acid extraction, suggesting this method has the prospect of wide application and can significantly improve the efficiency of RR-TB detection.

In recent years, several commercial molecular tests have been developed to determine the drug resistance of MTB isolates based on the detection of specific genetic mutations conferring resistance. Of these rapid tests, the GeneXpert (Cepheid, USA) has been endorsed by WHO for the detection of rifampicin (RIF) resistance (Pang et al., 2016). As to the simplicity of operation, GeneXpert is undoubtedly the most automatic platform for rapidly identifying RIF resistance. However, the Xpert equipment is costly and the limit of detection (LOD) of Xpert is only 131 CFU/mL. The loop mediated isothermal amplification (LAMP) can be also used for detecting drug resistant tuberculosis (Yadav et al., 2022). The LAMP method does not require special equipment and readily detects mutations in a quick manner, which meets the demand for developing point of care settings. However, the LAMP method requires complex primers and is not suitable for multiplex detection and the accuracy needs to be further improved (Takarada et al., 2020; Liu et al., 2021). The sensitivity of the MLP-RAP assay was 5 copies/μl in detecting DNA plasmids, which is 20 times more sensitive than qPCR (100 copies/μl). In addition, the MLP-RAP results using 78 boiled sputum samples achieved 100% concordance with the GeneXpert results. Meanwhile, the MLP-RAP can be performed on a conventional real-time PCR device that is more suitable for general common laboratories. Of note, after the addition of the extracted nucleic acid, the whole process of 96 samples could be completed within 1 h. The MLP-RAP offers a shorter turn-around time (1 h) to generate diagnostic results than GeneXpert (2.5 h). In addition, the MLP-RAP assay (4.5 $ per reaction) provides the lower test price for detecting RIF resistance–a tenth that of GeneXpert (45 $ per reaction). Therefore, the MLP-RAP assay is also a cost-effective method and has important potential for improving the diagnosis and control of RR-TB.

Many studies reported that the clinical samples had mixed infections containing mixtures of RIF-susceptible and RIF-resistant TB, and studies of these mixed samples are warranted (van Rie et al., 2005). The Xpert MTB/RIF assay could detect the presence of resistance when the mixture contained at least 65.6% mutant DNA (Blakemore et al., 2010). The MLP-RAP assay was designed to detect four common mutations associated with RIF resistance in the MUT tube, including D516V (GAC→GTC), H526D (CAC→GAC), H526Y (CAC→TAC), and S531L (TCG→TTG) and was able to detect 5% mutations in the genomic template containing 5 × 10^2^ genomes/μl. Analysis of clinical isolates confirmed that the MLP-RAP assay detected more heteroresistant samples than the Xpert MTB/RIF assay. Hence, given the practically high prevalence of heteroresistance in China, the better capability of MLP-RAP to detect mixed infection may facilitate laboratory staff to identify more RIF-heteroresistant TB cases at the early stage of the TB.

Nonetheless, the MPL-RAP assay has some limitations. First, the MPL-RAP analysis result cannot quantify the bacteria titer in the original samples. A special software needs to be developed to accurately analyze the results of rifampicin heteroresistance and give the percentage of drug resistance. Second, this assay only detects the mutation types at codons 516, 526, 531, and 533, not all codons of RRDR. Third, this study only tested 41 rifampicin-sensitive sputum samples, and more rifampicin-resistant sputum samples are needed to verify this method.

The emergence of drug-resistant TB—especially multidrug-resistant TB (MDR-TB, defined as resistance to at least isoniazid and rifampicin) and extensively drug-resistant TB (XDR-TB, defined as MDR-TB plus resistance to any fluoroquinolone and kanamycin, amikacin, or capreomycin)—is considered the greatest obstacle to global TB control due to difficulties in diagnosis and treatment (Wang et al., 2022). With the distinctive advantages of high sensitivity, rapidity, cost-effectiveness, and generality, we intend to extend the MLP-RAP strategy to other mutations associated with other drug resistance in TB. Ultimately, we attempt to integrate the MPL-RAP assay with microfluidic devices (Lutz et al., 2010) to achieve multiple and simultaneous screening of multidrug-resistant (MDR) and extensively drug-resistant tuberculosis (XDR-TB) patients from sputum samples.

## Data availability statement

The original contributions presented in this study are included in the article/Supplementary material, further inquiries can be directed to the corresponding authors.

## Ethics statement

All aspects of this study were performed by the National Ethics Regulations and approved by the Institutional Review Boards of the National Institute for Viral Disease Control and Prevention, Center for Disease Control, China. Written informed consent was obtained from the patients after informing them of the use of data for analysis and using the results for improving patient care activities and without disclosing their names or identity.

## Author contributions

XM, YZ, and XSh designed the study. RZ, XO, and XSu performed the experiments. XO and BZ collected the sample. GF, FT, and FL analyzed and interpreted the data. All authors provided critical review and approved the manuscript.

## Supplementary material

Supplementary Figure 1 Sensitivity of the MLP-RAP assay using diluted wild-type plasmid for eight replicates (10^6^, 10^5^, 10^4^, 10^3^, 10^2^, 10, and 5 copies/μl). In WT tube **(A)**, WT1 channel **(B)**, WT2 channel **(C)**, and WT3 channel **(D)** have signals. In the MUT tube (not shown), all channels have no signal. The sensitivity of the WT1 channel, WT2 channel, and WT3 channel could reach 5 copies/μl.

Supplementary Figure 2 Sensitivity of the MLP-RAP assay using diluted D516V plasmid for eight replicates (10^6^, 10^5^, 10^4^, 10^3^, 10^2^, 10, and 5 copies/μl). In WT tube **(A)**, WT2 channel **(B)** and WT3 channel **(C)** have signals. In MUT tube **(D)**, MUT1 channel **(E)** has a signal. The sensitivity of the WT2 channel, WT3 channel, and MUT1 channel could reach 5 copies/μl.

Supplementary Figure 3 Sensitivity of the MLP-RAP assay using diluted H526D plasmid for eight replicates (10^6^, 10^5^, 10^4^, 10^3^, 10^2^, 10, and 5 copies/μl). In WT tube **(A)**, WT1 channel **(B)** and WT3 channel **(C)** have signals. In MUT tube **(D)**, MUT2A channel **(E)** has a signal. The sensitivity of the WT1 channel, WT3 channel, and MUT2A channel could reach 5 copies/μl.

Supplementary Figure 4 Sensitivity of the MLP-RAP assay using diluted H526Y plasmid for eight replicates (10^6^, 10^5^, 10^4^, 10^3^, 10^2^, 10, and 5 copies/μl). In WT tube **(A)**, WT1 channel **(B)** and WT3 channel **(C)** have signals. In MUT tube **(D)**, MUT2B channel **(E)** has a signal. The sensitivity of the WT1 channel, WT3 channel, and MUT2B channel could reach 5 copies/μl.
