# Supplementary figures and images for: Multiplex LNA probe-based RAP assay for rapid and highly sensitive detection of rifampicin-resistant Mycobacterium tuberculosis
Source: Front Microbiol. 2023 Apr 27;14:1141424. doi: 10.3389/fmicb.2023.1141424 (PMC10172479; doi:10.3389/fmicb.2023.1141424)

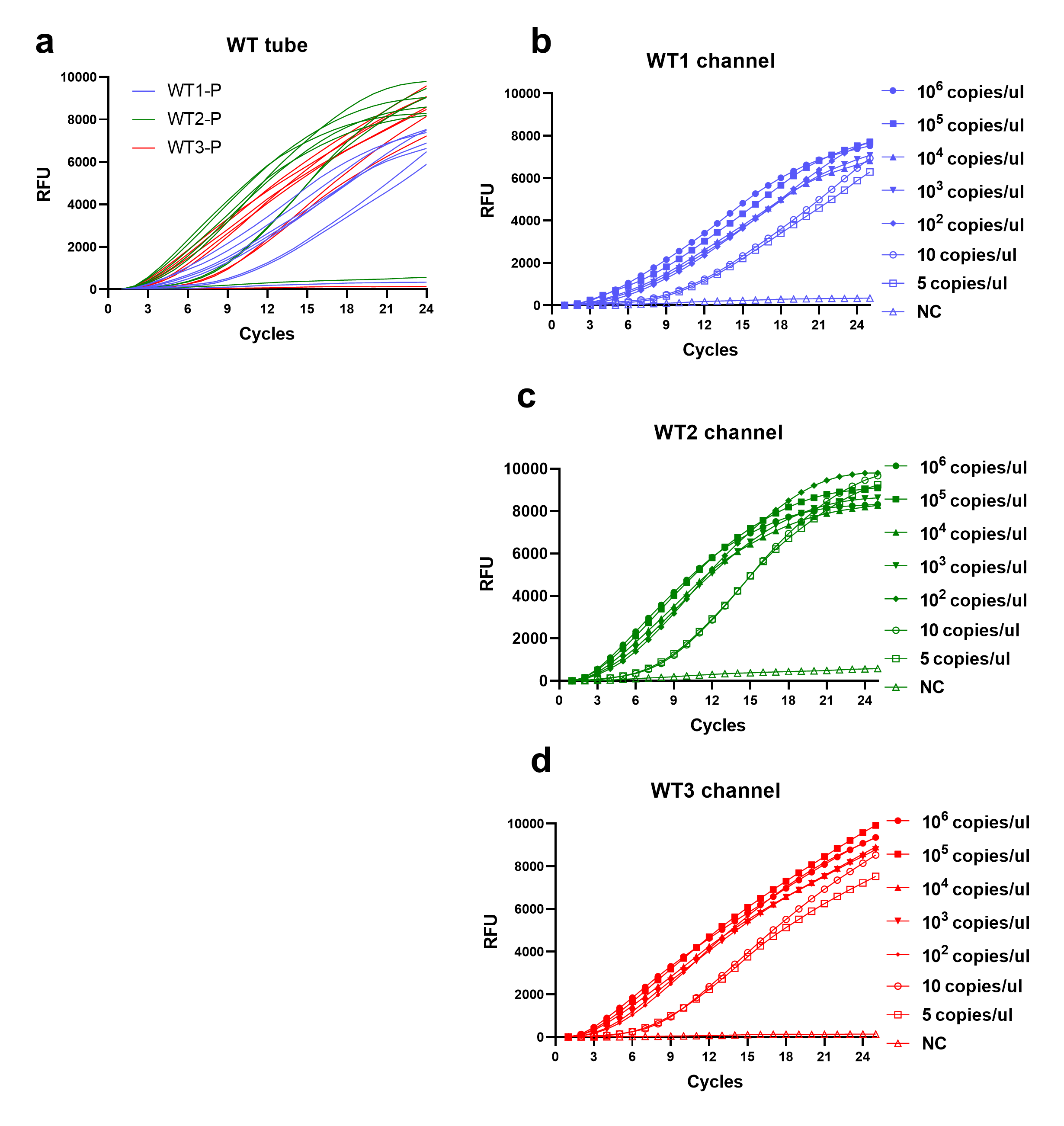

Supplement: Supplementary Figure 1 — Sensitivity of the MLP-RAP assay using diluted wild-type plasmid for eight replicates (106, 105, 104, 103, 102, 10, and 5 copies/μl). In WT tube (A), WT1 channel (B), WT2 channel (C), and WT3 channel (D) have signals. In the MUT tube (not shown), all channels have no signal. The sensitivity of the WT1 channel, WT2 channel, and WT3 channel could reach 5 copies/μl. [file Image_1.TIF]

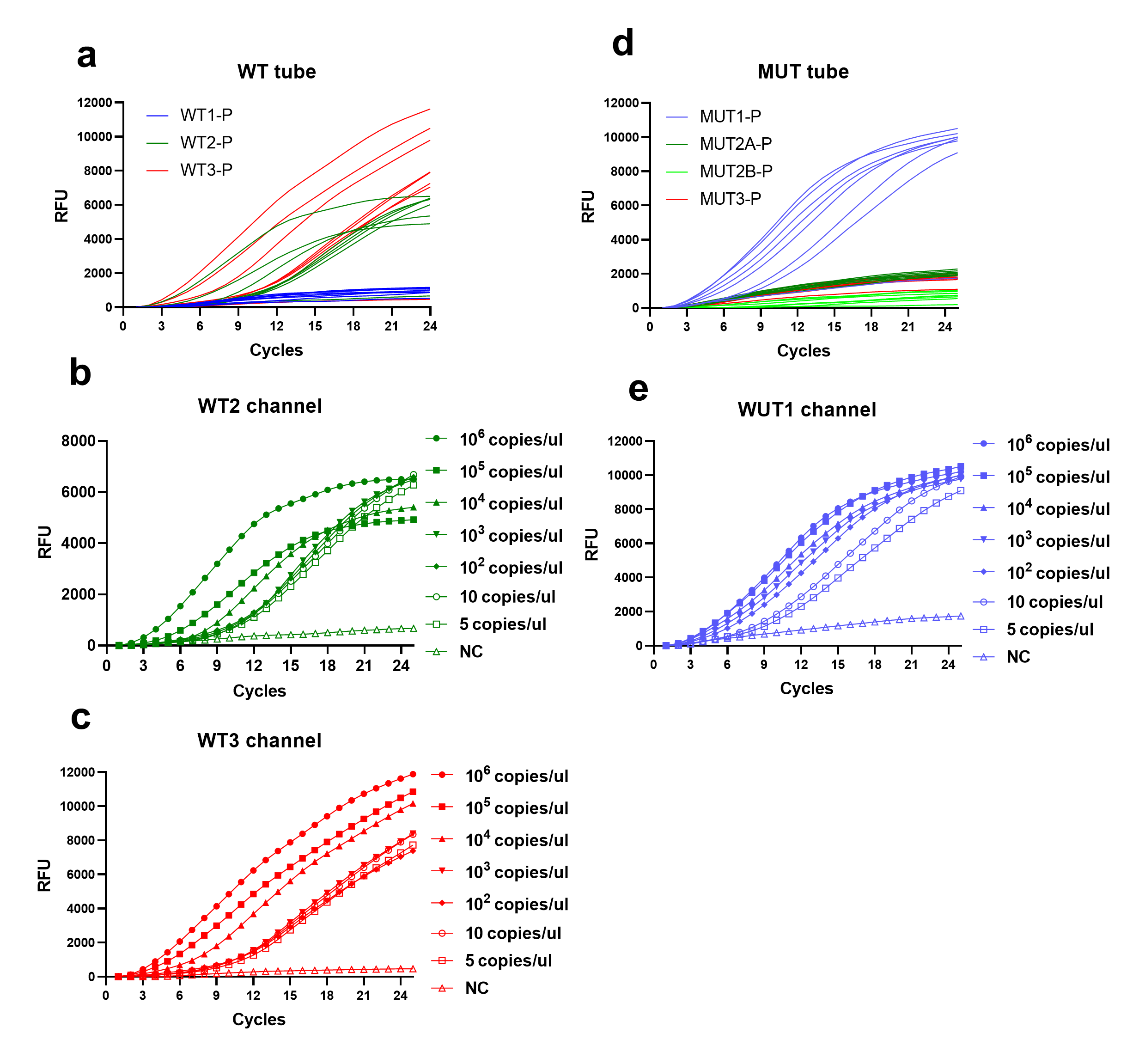

Supplement: Supplementary Figure 2 — Sensitivity of the MLP-RAP assay using diluted D516V plasmid for eight replicates (106, 105, 104, 103, 102, 10, and 5 copies/μl). In WT tube (A), WT2 channel (B) and WT3 channel (C) have signals. In MUT tube (D), MUT1 channel (E) has a signal. The sensitivity of the WT2 channel, WT3 channel, and MUT1 channel could reach 5 copies/μl. [file Image_2.TIF]

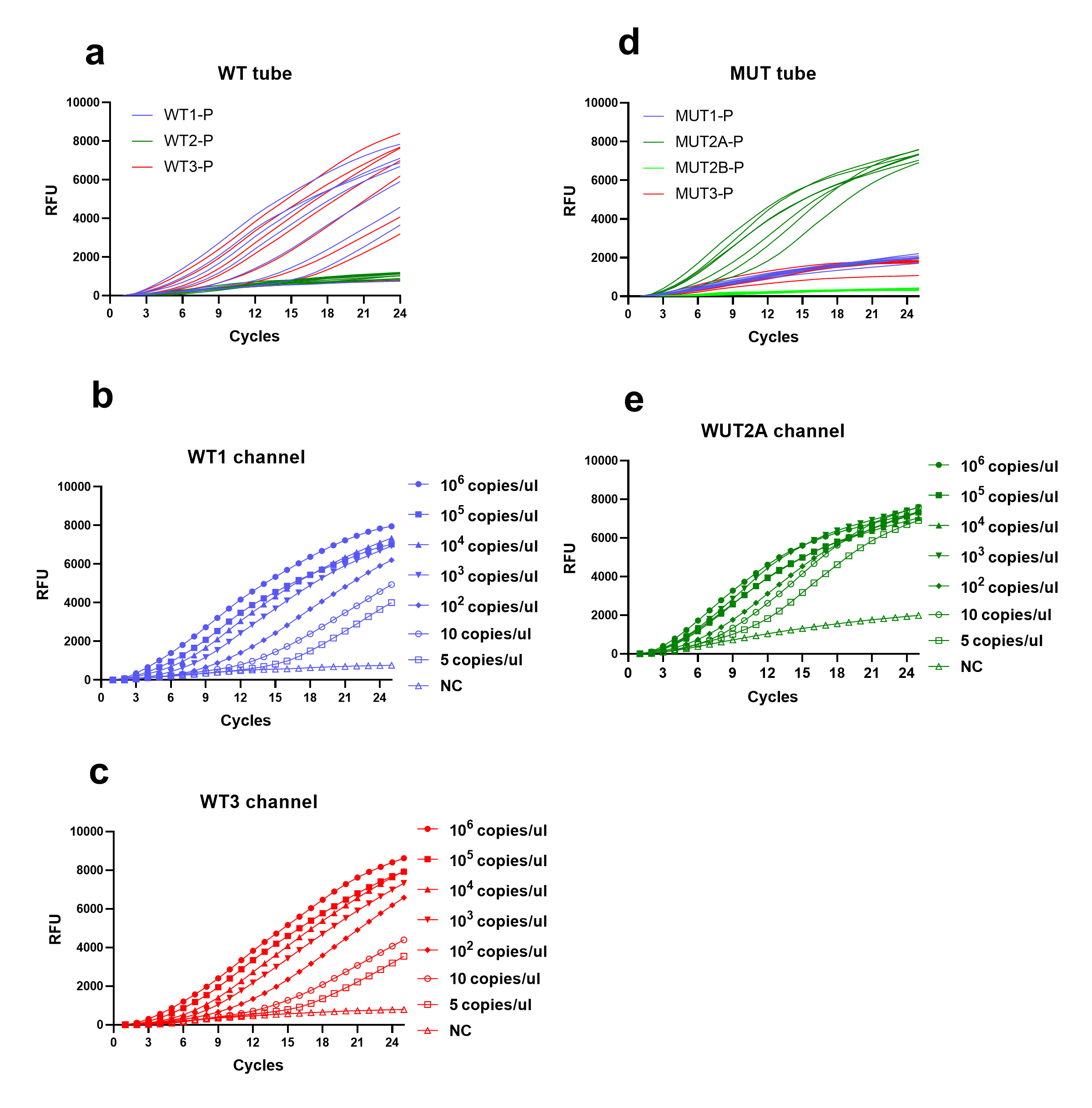

Supplement: Supplementary Figure 3 — Sensitivity of the MLP-RAP assay using diluted H526D plasmid for eight replicates (106, 105, 104, 103, 102, 10, and 5 copies/μl). In WT tube (A), WT1 channel (B) and WT3 channel (C) have signals. In MUT tube (D), MUT2A channel (E) has a signal. The sensitivity of the WT1 channel, WT3 channel, and MUT2A channel could reach 5 copies/μl. [file Image_3.TIF]

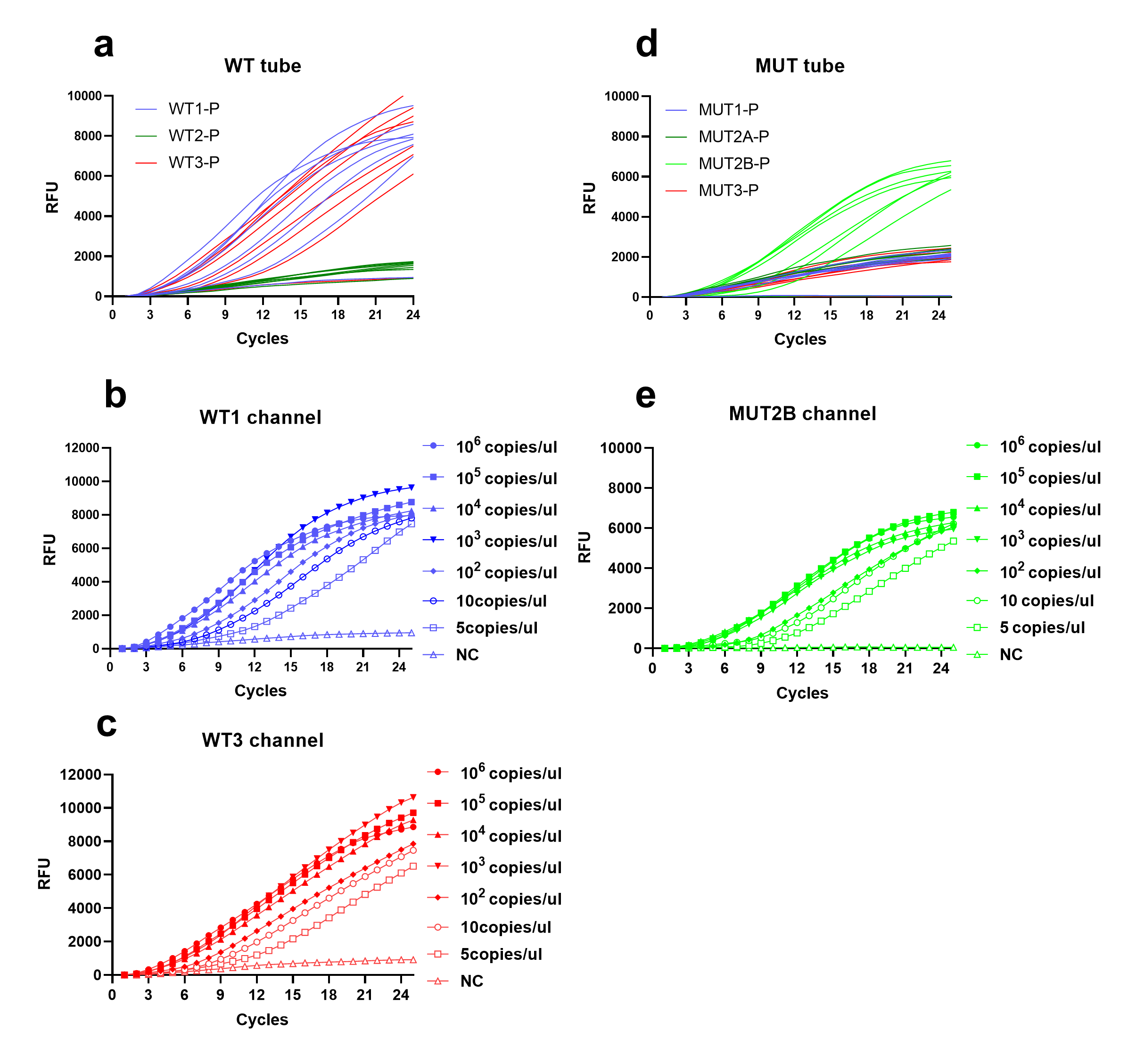

Supplement: Supplementary Figure 4 — Sensitivity of the MLP-RAP assay using diluted H526Y plasmid for eight replicates (106, 105, 104, 103, 102, 10, and 5 copies/μl). In WT tube (A), WT1 channel (B) and WT3 channel (C) have signals. In MUT tube (D), MUT2B channel (E) has a signal. The sensitivity of the WT1 channel, WT3 channel, and MUT2B channel could reach 5 copies/μl. [file Image_4.TIF]
